# The era of advanced therapeutics for pediatric atopic dermatitis – can early systemic intervention reduce the type 2 inflammatory response and modify the atopic march?

**DOI:** 10.1097/MOP.0000000000001576

**Published:** 2026-05-07

**Authors:** Florence Vroman, Marlies de Graaf

**Affiliations:** Department of Dermatology, University Medical Center Utrecht, University of Utrecht, Utrecht, The Netherlands

**Keywords:** atopic dermatitis, atopic march, biologics, disease modification

## Abstract

**Purpose of review:**

The recent development of advanced systemic treatment options for pediatric atopic dermatitis (AD) means that achieving long-term, off-therapy remission, so-called disease modification, has become a subject of discussion. Emerging evidence suggests that early intervention during a potential ‘window of opportunity’ could alter the natural course of AD. If such a window could be identified, early and targeted treatment might induce long-term disease remission and might reduce the risk of the development of highly burdensome atopic comorbidities.

**Recent findings:**

Among currently available therapies, dupilumab, targeting interleukin (IL)-4 and IL-13 signaling, provides the most compelling evidence for potential disease modification. Studies indicate that a subset of patients treated with dupilumab may achieve prolonged remission after treatment discontinuation, and that treatment may reduce the risk of subsequent allergic disease development.

**Summary:**

Disease modification and long-term remission are no longer an idle hope for AD patients. However, translating this into clinical practice remains challenging due to the heterogeneity of AD and the lack of consensus of definitions. Future research should therefore focus on establishing these definitions, and on determining whether early systemic intervention can truly modify the disease itself and the atopic march.

## INTRODUCTION

Atopic dermatitis (AD) is a chronic inflammatory skin disease that typically presents in early childhood but often persists into adulthood [1]. It is characterized by a complex interplay of genetic predisposition, environmental triggers, impaired skin barrier function, microbial dysbiosis, and T-helper (Th)2-driven immune dysregulation [2]. Disease course is highly variable and although predicting a more prolonged AD course remains challenging, clinically relevant predictors such as disease severity, age at AD onset, family history of atopy, allergic sensitization, loss-of-function filaggrin (*FLG*) mutations, and urban upbringing have been identified [3,4,5^▪^]. Among these factors, early disease severity emerges as a potentially modifiable factor through timely and appropriate therapeutic intervention. Given the profound impact of AD on the quality of life of patients and their families, early intervention with the potential for disease modification remains an important therapeutic goal [1,6]. Moreover, as AD is often considered the first manifestation of the atopic march, early intervention may provide an opportunity to alter the natural course of further development of allergic diseases [7]. 

**Box 1:**
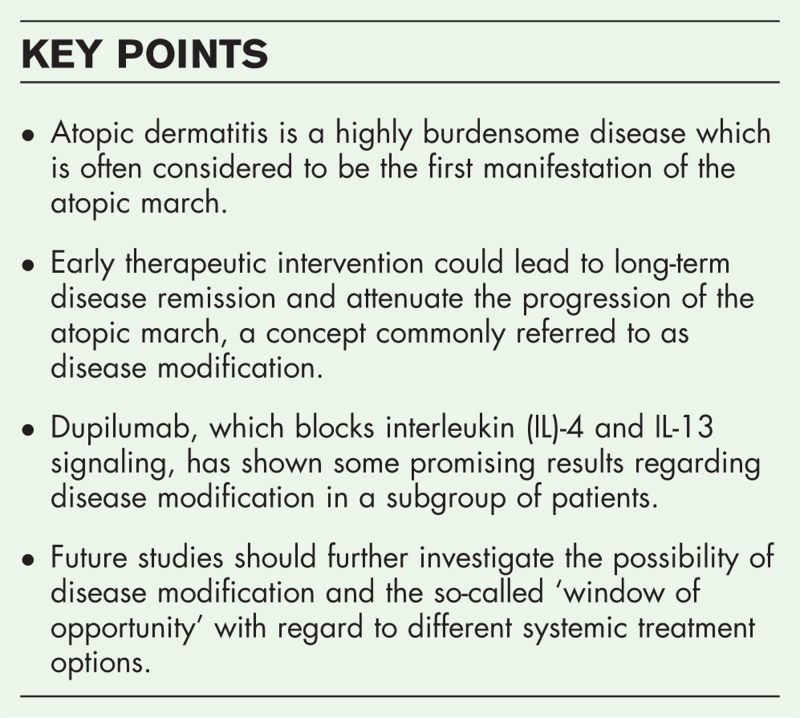
no caption available

## ATOPIC MARCH

AD is often regarded as the first manifestation of the atopic march, which refers to the sequential development of different allergic diseases during childhood [7,8]. The atopic march develops in approximately two-thirds of children with AD, with a higher risk observed in those who produce immunoglobulin (Ig) E antibodies in response to environmental triggers [9^▪^]. AD is often followed by the development of other atopic comorbidities, including food allergies (FA), asthma, and allergic rhinitis (AR) [10–13]. The progression of the atopic march is thought to result from a complex interplay between impaired skin barrier function, increased *Staphylococcus aureus* abundance, and Th2-driven immune dysregulation [5^▪^,^14^]. As described by Bieber, the immunological process can be dissected into several potentially overlapping phases: (i) an asymptomatic preclinical phase; (ii) activation of skin innate immunity; and (iii) subsequent activation of the adaptive immune response. Initially, these processes are dominated by a core Th2 response with IgE sensitization to environmental allergens, followed by the widening of the adaptive immunity with Th17, Th22, and finally Th1 responses [7]. This widening paves the way for the development of atopic comorbidities, with these immunological processes persisting despite clinical remission of AD. Indeed, spontaneous remission is common in AD, with 40% of patients presenting with early-onset AD being in remission by the age of three [15^▪▪^]. Although AD may enter spontaneous long-term remission, the mechanisms leading to the atopic march may have already been activated [7,16].

## DISEASE MODIFICATION IN ATOPIC DERMATITIS

**FIGURE 1:**
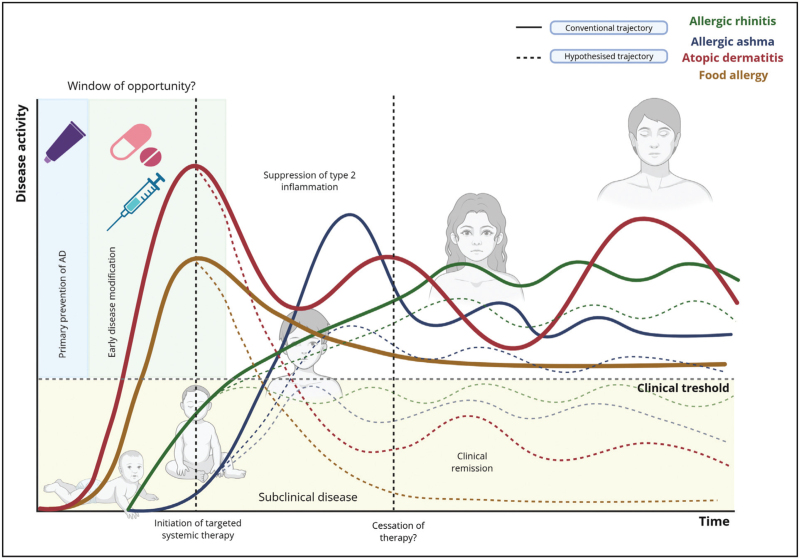
This figure demonstrateds the concept of disease modification in pediatric atopic dermatitis showing the window of opportunity for early systemic intervention to modify/attenuate the atopic march. Atopic dermatitis typically arises early in life and may precede the development of other type 2 inflammatory diseases, including food allergy, asthma, and allergic rhinitis. Early initiation of targeted systemic therapy during a ‘*window of opportunity*’ may suppress type 2 inflammation, limit immune imprinting, and attenuate progression of the atopic march, potentially leading to disease modification. The horizontal dashed line represents the clinical threshold above which disease becomes clinical apparent. Solid lines represent the conventional disease trajectories, whereas dotted lines indicate hypothesized attenuated trajectories following early intervention. Outcomes following treatment cessation remain uncertain, as therapies may exert disease-modifying effects that could result in persistent clinical remission. These potential effects require confirmation in future studies. Figure created with BioRender.com.

An emerging hypothesis suggests that early therapeutic intervention could endurably impact the pathomechanisms and the natural course of AD by altering the underlying Th2-mediated pathways leading to sustained remission and attenuation of the atopic march. This concept is commonly referred to as disease modification (DM). DM is well established in diseases such as rheumatoid arthritis (RA)/juvenile idiopathic arthritis (JIA) and inflammatory bowel diseases, where the tissue damage is irreversible and the aim of DM lies in slowing down the disease progression [15^▪▪^,^17^,^18^]. However, as there is no structural damage leading to deformation or scarring in AD, the concept of DM in AD is not well defined [6]. For the definition of DM in inflammatory skin disorders, two main aspects must be clearly distinguished: (i) the impact on the disease itself, (i.e. the chronic inflammation in the skin), and (ii) the impact on the associated comorbidities, (i.e. the atopic march) [7]. With this in mind, DM in AD can be possibly defined as therapeutic interventions that alter the natural course of the disease, potentially preventing or delaying the progression to a more severe disease, thereby achieving prolonged remission or sustained reduction in disease severity after treatment discontinuation, and/or reducing the risk of new-onset AD-related comorbidities, with or without ongoing treatment [6,19]. Indeed, the goal of DM is to prevent ongoing inflammation, alter the natural course of AD, and thereby hamper the progression of atopic comorbidities (Fig. 1) [7,20].

Increasing evidence shows that early-onset (<2 years) AD poses the highest risk of developing asthma, which is one of the most burdensome atopic comorbidities [7,13]. Additionally, Lin *et al.* identified risk factors for the development of the atopic march in children diagnosed with AD at <1 year of age which included male sex, severe AD, family history of atopy, cesarean section delivery, and maternal antibiotic use during pregnancy [12]. Furthermore, infants and young children have developing immune systems and are potentially more susceptible to DM. Therefore, early-life initiation of targeted therapies is of particular interest, given their potential to halt the progression of the atopic march (Fig. 1).

Currently, no therapies for AD have been recognized as disease-modifying by regulatory agencies. Previous studies have focused on prevention efforts, including skin barrier (emollient) interventions, but results are inconsistent [21,22]. The STOP-AD trial showed a statistically significant reduction in AD incidence at the age of 12 months following short-term use of a emollient from birth to 2  months in high-risk infants. On the other hand, results from the BEEP randomized trial showed that daily emollient application during the first year of life did not prevent AD at the age of 3–5 years [22,23]. Further research is needed to identify the potential preventive role of emollient use in AD onset and to determine whether it is truly about prevention rather than just delaying the development of AD. Additionally, pimecrolimus, a calcineurin inhibitor, was evaluated for its effects on the atopic march when administered at the first manifestation of AD in infancy by assessing its effect on asthma incidence. However, no significant difference was found between the treated and non-treated group in terms of asthma development [8,24]. Nevertheless, studies on the use of topical corticosteroids (TCS) have shown that proactive treatment may lead to a lower risk of developing a FA compared to placebo. This suggests that the promptness with which AD patients are treated can influence both the course of the disease and the development of allergic comorbidities, the so called ‘window of opportunity’ [4,12,25–27].

## WINDOW OF OPPORTUNITY

Given the central role of type 2 immune pathways in the pathogenesis of the atopic march, recent focus has shifted towards therapies targeting these pathways with systemic treatment options as potential disease-modifying interventions. Promising results from biologics are demonstrated in other chronic inflammatory diseases, such as psoriasis and RA/JIA, where the timing of initial treatment can significantly impact the long-term disease outcomes [7,15^▪▪^,^18^,^28^]. In psoriasis, biologics have shown potential for DM by showing extended remission periods after treatment withdrawal and a lower risk of developing psoriatic arthritis in adults [4].

The question remains whether this is also the case for AD and whether it would be possible to stop the atopic march and to determine what could be the optimal timing for intervention, the so called ‘window of opportunity’ (Fig. 1). It is assumed that there is a window of time at the very early stages of AD during which DM may be possible, but it is unclear when and how long this window lasts [29,30]. To date, the effect of targeted therapies on disease progression and development of comorbidities in AD remains poorly understood.

## AVAILABLE ADVANCED SYSTEMIC TREATMENT OPTIONS FOR PEDIATRIC ATOPIC DERMATITIS

Currently, both biologics (dupilumab, tralokinumab, and lebrikizumab) and Janus kinase (JAK) inhibitors (baricitinib, abrocitinib, and upadacitinib) are available for treating pediatric AD, of which the majority is only approved from the age of 12  years (Table 1). Expansion of market authorization to younger patients is expected for some of these treatments since clinical trials with tralokinumab (NCT05388760, NCT06311682), lebrikizumab (NCT05559359), upadacitinib (NCT06701331), and abrocitinib (NCT06807281) are currently running or planned. Additionally, new therapies have been developed, such as OX40 inhibitors (NCT05769777, NCT05882877), but their efficacy and safety still needs to be determined, especially in the pediatric population.

**Table 1:** Overview of advanced systemic therapies in pediatric atopic dermatitis and their potential disease-modifying effects

Therapy class	Target/pathway	Pediatric approval^*^	Evidence suggesting disease modification	Key evidence gaps
IL-4/IL-13 inhibition (e.g., dupilumab)	Inhibition of the IL-4Rα leading to inhibition of IL-4 and IL-13 signaling	≥6 months	Long-term disease control, reduction in type 2 biomarkers, (s)IgE decline, signals of sustained remission after discontinuation, potential reduction in development of new atopic comorbidities	Lack of prospective trials designed to demonstrate disease modification; sustained remission after withdrawal insufficiently investigated
Selective IL-13 inhibitors (e.g., tralokinumab, lebrikizumab)	IL-13 neutralization	≥12 years	Improvement in barrier function and sustained disease control; mechanistic rationale for modifying type 2 inflammation	Limited pediatric long-term data; unclear impact on atopic march; limited withdrawal data
JAK inhibitors (e.g., baricitinib, upadacitinib, abrocitinib)	Inhibition of JAK-STAT pathway leading to broad cytokine inhibition	Abrocitinib and upadacitinib ≥12 years; baricitinib ≥2 years	Rapid suppression of multiple inflammatory pathways, theoretical potential to alter inflammatory set-point	Limited pediatric long-term data; No evidence for disease modification or impact on the atopic march; limited withdrawal data
OX40/OX40L inhibitors (e.g., rocatinlimab, amlitelimab)	Modulation of activated T cells and reduction of T cell proliferation	Not approved	Theoretical potential to affect immune memory and induce sustained remission	Early-phase evidence; pediatric data lacking; impact on atopic march unknown

^*^Approval status differs across regions.

(s)IgE, (specific) immunoglobulin E; e.g., exempli gratia; IL, interleukin; IL-4Rα, interleukin 4 receptor alpha; JAK, Janus kinase; OX40L, OX40 ligand.

### Biologics

Recent literature suggests that dupilumab is a promising disease-modifying treatment option [6,9^▪^,^11^]. It is a monoclonal antibody that targets the interleukin (IL)-4 receptor alpha, thereby inhibiting both IL-4 and IL-13 signaling, which are key drivers of the Th2 response. Dupilumab was the first systemic treatment option approved for pediatric AD and is now approved from the age of 6  months by the Food and Drug Administration (FDA) and European Medicines Agency (EMA) [31]. Clinical trials and real-world studies have shown the effectiveness and safety of dupilumab in treating pediatric AD patients [32–35]. Notably, dupilumab treatment in AD patients improves skin barrier function, decreases *S. aureus* abundance, and reduces biomarkers of type 2 inflammation, such as serum IgE [36–39]. Furthermore, dupilumab is approved for other type 2-mediated diseases, including asthma, highlighting its possible immunomodulatory effect [40]. Indeed, dupilumab appears to influence the new acquisition and/or worsening of atopic comorbidities in adolescents. Geba *et al.* conducted a meta-analysis of data from clinical trials and reported a 34% reduction in new allergies in patients over 12  years old, which persisted even during off-treatment periods [41]. These results are further supported by real-world evidence. Lin *et al.* showed that patients on dupilumab treatment had a significant decreased risk of developing the atopic march towards asthma or AR (32%) compared with patients on conventional systemic therapy [12]. This effect was even more pronounced in younger children, highlighting the importance of the window of opportunity. Similarly, the study of Tsai *et al.* showed that the treatment with dupilumab compared to systemic agents resulted in a reduction of AD-related comorbidities (such as asthma and AR) in pediatric patients, with more pronounced effects in younger children (aged 0–5  years) [42]. Furthermore, dupilumab also seems to have a positive effect on existing atopic comorbidities as previous studies have shown that food- and aeroallergen-specific IgE levels in patients with FA, asthma, and/or AR significantly decreased during dupilumab treatment [43,44]. Another interesting aspect of dupilumab is the possibility of AD remission after treatment discontinuation. Remission, which is defined as a sustained improvement or resolution of disease symptoms and signs, has been demonstrated in adults treated with dupilumab, for up to 40 weeks after treatment cessation [9^▪^,^45^]. Earlier presentation of preliminary data from pediatric patients showed that approximately one-third of patients achieved clinical remission, with half of them remaining therapy-free [9^▪^]. Based on these studies, dupilumab positively impacts AD and seems to have the potential to impact the atopic march, which aligns with the previous definition of DM. However, future studies are needed to confirm its disease-modifying potential.

Tralokinumab and lebrikizumab, both IL-13 inhibitors, are only approved from the age of 12  years. Although current evidence on their effect on the atopic march is limited, they are likely to contribute to the attenuation of the atopic march due to their effect on the skin barrier repair [11]. Nevertheless, previous studies found no significant improvement in moderate-to-severe asthma by single IL-13 inhibition, suggesting that IL-4 inhibition plays a more crucial role in the atopic march [46]. The question arises whether these IL-13 inhibitors show the same potential regarding AD remission as dupilumab. Results from lebrikizumab follow-up studies seem to hint at this as EASI90 response was sustained in a subgroup of patients up to 38  weeks after lebrikizumab withdrawal [47]. However, future studies are needed to assess the exact impact of these treatment options on the atopic march [6,46].

### Janus kinase inhibitors

JAK inhibitors target multiple pathways involved in AD by blocking the activity of the JAK family enzymes and subsequently disrupting cytokine signaling [9^▪^]. Baricitinib, a JAK1/2-selective inhibitor, has been approved by the EMA for the treatment of AD in pediatric patients from the age of 2  years, while other available JAK inhibitors, including abrocitinib and upadacitinib, are approved from the age of 12  years [48,49,50]. Although there is currently no evidence, JAK inhibitors may have potential for DM based on their mode of action. The JAK-STAT pathway leads to signaling of type 2 cytokines, including IL-4, IL-13, IL-31, and thymic stromal lymphopoietin (TSLP). These cytokines contribute to chronic inflammation and immune dysregulation in AD. By inhibiting the JAK–STAT pathway, the underlying mechanisms resulting in immune dysregulation might be disrupted. As a result, early intervention with a JAK inhibitor in AD patients may prevent long-term immune changes and potentially alter the course of AD [6]. However, to date, no studies have demonstrated a positive effect of JAK inhibitors on allergic disease outcomes in AD patients, nor their potential for disease-modifying effects. Therefore, further research is needed to confirm this.

### OX40 inhibitors

Currently, OX40 inhibitors are being investigated as potential treatment options for AD. The OX40-OX40 ligand (OX40L) interaction is involved in the activation of CD4^+^ T cells and promotes the expansion and survival of Th2 cells [5^▪^]. Additionally, the interaction indirectly enhances B cell activation and IgE class switching, primarily through upregulated T follicular helper cell responses [51,52]. Moreover, OX40-OX40L signaling is critical for the formation of memory T cells, thereby contributing to persistent disease. Rocatinlimab and amlitelimab both target the OX40-OX40L interaction, with rocatinlimab targeting the OX40 receptor and amlitelimab the OX40L. Both treatments target the earlier stages of the immunological cascade and may therefore also show disease-modifying potential [9^▪^]. In fact, promising results were found for rocatinlimab as AD improvement in adult patients continued through 36  weeks of active treatment, which was largely maintained in responders throughout a subsequent 20-week off-treatment period [53]. Similar results were found for amlitelimab, where a subgroup of good responders was shown to have a sustained response up to 24 weeks after drug withdrawal [54]. These data suggest that by targeting the pathogenic T cells directly, T cell number and activity might rebalance and lead to a durability of response following discontinuation of these treatments. Data in children are lacking and the effects of OX40 inhibitors on the atopic march remain largely unknown.

## FUTURE PERSPECTIVES

Defining DM in AD remains challenging, and future studies are needed to establish clear assessment methods. However, this is complicated by the variable course of AD and its high rate of spontaneous remission, particularly in young children. Therefore, future research should aim to determine the optimal timing for treatment cessation, as well as clarifying whether sustained improvement reflects true DM or natural disease remission. Epigenetic studies are promising, as recent evidence shows that dupilumab has a distinct epigenetic effect on T cells of adult AD patients, further supporting its disease-modifying potential [55^▪^]. Combining clinical and translational research could improve our understanding of DM in AD and its potential impact on the development of atopic comorbidities.

## CONCLUSION

The therapeutic landscape for pediatric AD patients has evolved tremendously in recent years, and the goal of achieving long-term, off-therapy remission has become more than a distant hope. As systemic treatment options become increasingly available at younger ages, their potential impact on the atopic march should be further investigated. Although future studies should investigate the potential of DM, defining this concept and determining the window of opportunity remains challenging. Nevertheless, once a clear definition has been established, the concept of DM could evolve from an emerging hypothesis to a defined therapeutic strategy in pediatric AD.

## Acknowledgements

*None.*

### Financial support and sponsorship

*None.*

### Conflicts of interest

*F. Vroman has been a speaker for Sanofi and M. de Graaf has been a consultant, advisory board member, and/or speaker for AbbVie, ALK, Almirall, Eli Lilly, Galderma, Janssen, LEO Pharma, Novartis, Pfizer, Regeneron Pharmaceuticals, and Sanofi.*

## REFERENCES AND RECOMMENDED READING

Papers of particular interest, published within the annual period of review, have been highlighted as:

▪ of special interest
▪▪ of outstanding interest
